# Deep learning-based automated detection of supernumerary teeth in pediatric panoramic radiographs

**DOI:** 10.1371/journal.pone.0335845

**Published:** 2025-11-05

**Authors:** İlhan Uzel, Behrang Ghabchi, Dilşah Çoğulu

**Affiliations:** Department of Pediatric Dentistry, Faculty of Dentistry, Ege University, Izmir, Turkey; Polytechnic University of Marche, ITALY

## Abstract

**Introduction:**

Supernumerary teeth are a common developmental anomaly in pediatric patients, potentially leading to complications such as impaction, crowding, and delayed eruption. Accurate and early detection is critical to prevent these sequelae and guide appropriate intervention strategies. This study aims to evaluate the diagnostic accuracy and clinical applicability of a convolutional neural networks-based deep learning model (YOLOv8) for the automated localization and binary classification of supernumerary teeth on pediatric panoramic radiographs.

**Materials and methods:**

A retrospective analysis was conducted on 2000 pediatric panoramic radiographs following ethical approval. Three calibrated pediatric dentists independently examined the dataset and annotated a representative subset of 140 radiographs (71 positive, 69 negative), achieving substantial inter-rater agreement (Cohen’s κ = 0.92). Performance was assessed in two stages: (1) segmentation of supernumerary teeth and (2) binary classification of radiographs. An independent validation set of 20 radiographs was used for secondary evaluation. Evaluation metrics included precision, recall, F1-score, and McNemar’s test to compare model predictions with expert labelling.

**Results:**

The mean age of the patients was 9.6 ± 2.3 years; 52% were male, 48% were female. The segmentation model yielded 100% precision, 38% recall, and an F1-score of 55%, indicating strong localization when detections were made but limited sensitivity. The classification model achieved 100% accuracy, precision, recall, and F1-score on both internal and external datasets. McNemar’s test revealed no statistically significant discrepancy between the model and expert decisions (p > 0.05). The segmentation model demonstrated high precision in localizing supernumerary teeth; however, recall performance was more modest, indicating occasional under-detection. Due to the limited validation sample size, these findings should be interpreted with caution.

**Conclusions:**

The YOLOv8-based pipeline demonstrated robust diagnostic accuracy in classifying panoramic radiographs for supernumerary teeth and promising but preliminary results in lesion-level segmentation. These findings highlight the potential utility of advanced deep learning systems in augmenting early diagnosis and streamlining pediatric dental radiology workflows.

## 1. Introduction

Supernumerary teeth are a developmental dental anomaly characterized by the presence of additional teeth beyond the normal dental formula [1]. They may be single or multiple, unilateral or bilateral, and can appear in either the maxilla or mandible. Epidemiological data suggest that these anomalies are up to ten times more prevalent in the maxilla, particularly in the premaxillary region, followed by the mandibular premolar area [1,2]. Supernumerary teeth can emerge in both primary and permanent dentitions, with a reported prevalence ranging from 0.1% to 3.8% depending on geographic and ethnic factors. This variability underscores the importance of region-specific studies to elucidate etiological patterns and refine diagnostic frameworks [3,4].

The clinical implications of undiagnosed supernumerary teeth include impaction, displacement, root resorption, cyst formation, and esthetic disturbances [5]. Although the etiology remains inconclusive, both genetic predisposition and syndromic associations have been implicated [3,4]. Accurate diagnosis requires a combination of clinical examination and radiographic imaging. Traditional modalities such as panoramic, occlusal, and periapical radiographs offer two-dimensional projections, whereas cone-beam computed tomography (CBCT) enables detailed three-dimensional localization and morphological assessment [2–4].

While many supernumerary teeth are asymptomatic and incidentally discovered, their timely detection is essential for optimal interceptive treatment and avoidance of long-term complications [5,6]. Panoramic radiography is the most commonly employed diagnostic modality due to its accessibility and broad coverage. However, its limited resolution in the buccolingual plane presents diagnostic challenges, especially for deeply impacted or poorly oriented teeth. CBCT improves locational resolution but is constrained by radiation concerns and cost [7].

Recent advances in artificial intelligence (AI), particularly deep learning algorithms, have enabled the automated analysis of dental radiographs for anomaly detection. These techniques hold promise in augmenting clinical workflows by enhancing diagnostic accuracy, consistency, and efficiency. Machine learning models especially convolutional neural networks (CNNs) can identify dental anomalies with a high degree of sensitivity and specificity across large image datasets [8].

Deep learning frameworks such as CNNs have demonstrated impressive performance in detecting supernumerary teeth on panoramic and CBCT scans, outperforming manual methods in speed and scalability [9,10]. These AI systems can precisely localize abnormal teeth, estimate morphological features, and support treatment planning. Despite their promise, existing studies are often constrained by limited datasets, inconsistent validation protocols, and restricted generalizability across populations. Furthermore, challenges in algorithm transparency, data labeling quality, and integration into routine clinical practice persist.

In contrast to previous work that focused on cropped regions of interest, this study uniquely analyzes full-field panoramic radiographs without pre-selection, thereby simulating a more realistic and complex diagnostic setting [11]. dditionally, unlike studies that emphasize classification alone, we implement a dual-stage model comprising both classification and segmentation to assess presence and positional localization simultaneously. This design better reflects clinical scenarios, particularly in pediatric patients with mixed dentition and high inter-individual variability [11,12].

Given the lack of consensus on optimal model architectures for this task, we employed YOLOv8, a state-of-the-art object detection and segmentation framework recognized for its real-time performance and adaptability to medical image analysis. This architecture offers a unified pipeline capable of detecting and delineating supernumerary teeth with minimal pre-processing, making it suitable for clinical implementation.

Accordingly, the aim of this study was to develop and validate a YOLOv8-based deep learning model for the automated detection and segmentation of supernumerary teeth on pediatric panoramic radiographs, and to assess its diagnostic performance in terms of accuracy, sensitivity, and generalizability.

## 2. Materials and methods

### 2.1. Study design and data collection

A retrospective design was adopted to assemble a representative dataset of pediatric panoramic radiographs acquired between January 2022 and December 2023. Radiographs were eligible for inclusion if they were obtained from patients aged 6–13 years and displayed complete maxillary and mandibular arches with diagnostically acceptable image quality.

Exclusion criteria comprised:

- Significant motion artifacts or poor contrast resolution
- Incomplete anatomical coverage
- Presence of metallic or structural artifacts likely to interfere with manual labeling or model predictions

This sampling method allowed for a complete and representative collection of pediatric dental radiographs within the study timeframe, maximizing internal validity while ensuring annotation quality for deep learning model development.

After excluding images, a total of 2000 anonymized digital panoramic radiographs were included. Ethical approval was granted by the Ege University Ethics Committee (Ref No: 23-11.1T/50), and written informed consent was obtained from parents or guardians, in compliance with institutional and international ethical guidelines.

To standardize the dentition stage and minimize inter-individual developmental variability, the age range of 6–13 years was chosen to reflect the mixed dentition period, optimizing clinical homogeneity for deep learning training.

### 2.1. Dataset construction

Three calibrated pediatric dentists independently reviewed the anonymized dataset to identify cases exhibiting supernumerary teeth, achieving high inter-rater agreement (Cohen’s κ = 0.92). A subset of 140 radiographs (71 positive, 69 negative) was curated for model development and evaluation.

For segmentation tasks, 71 radiographs containing at least one supernumerary tooth were manually annotated using a polygonal segmentation approach via CranioCatch (Eskişehir, Türkiye). Each supernumerary tooth was delineated at the instance level, ensuring anatomical precision.

**Fig 1:**
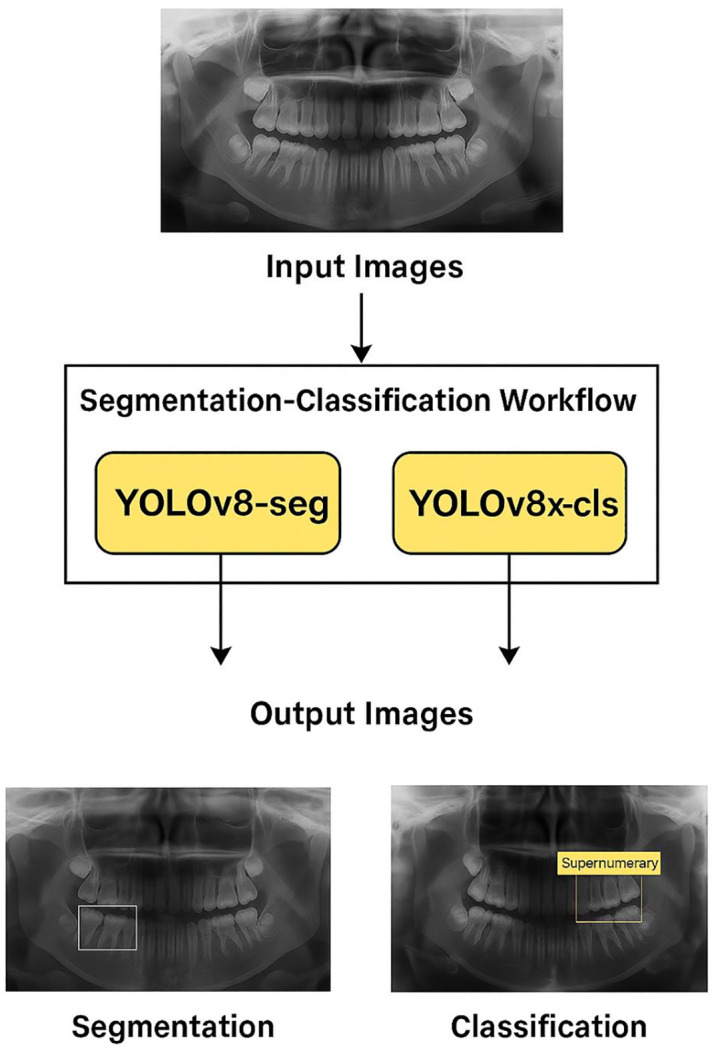
Flowchart of the study design.

For the classification task, an additional 69 radiographs without supernumerary teeth were included to form a balanced dataset of 140 radiographs. The dataset was divided into training, validation, and test subsets (Table 1, Fig 1). The independent validation set consisted of 20 radiographs from different patients not included in training or internal validation sets. These images were captured using the same imaging equipment but were temporally and operationally distinct, thereby simulating external testing conditions.

**Table 1:** Combined dataset overview for segmentation and classification tasks.

Task Type	Subset Type	Number of Radiographs
**Segmentation**	Training Dataset	57
	Validation Dataset	7
	Test Dataset	7
	Total	71
**Classification**	Training Dataset	112
	Validation Dataset	14
	External Test Set	14
	Total	140

Summary of the dataset distribution across segmentation and classification tasks. Segmentation was performed on a manually annotated subset of 71 radiographs with confirmed supernumerary teeth. Classification was conducted on a balanced dataset of 140 radiographs (71 with, 69 without supernumerary teeth), including an independent external test set for generalizability evaluation.

All radiographs were anonymized, and no personal identifiers such as name, gender, age, address, or contact information were used. Three calibrated pediatric dentists independently assessed the anonymized dataset to identify the presence of supernumerary teeth, resulting in a prevalence of 3.6% (n = 71/2000).

To further evaluate model generalizability, an independent validation set was constructed using radiographs from a separate patient cohort acquired at least three months after the training dataset. Although the same imaging device (Planmeca ProMax 2D, Planmeca Oy, Helsinki, Finland) was used, all patients in the external set were unique, and the temporal separation minimized the risk of data leakage. Random sampling was applied to reflect natural anatomical and clinical variation does not present in the original training set.

All images were acquired in JPEG format to preserve high-resolution input compatible with deep learning analysis. Preprocessing steps included resizing and intensity normalization to standardize the data for model training.

### 2.3. Deep learning architecture and training

**Fig 2:**
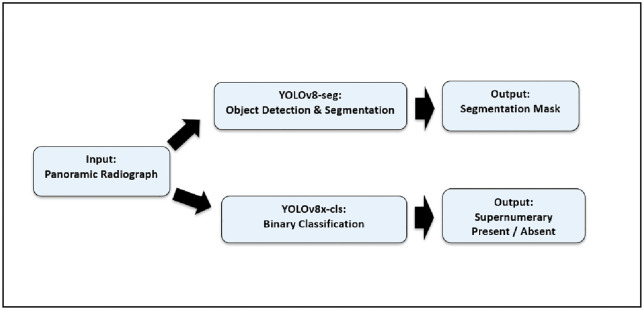
YOLOv8 architecture.

The YOLOv8 architecture, implemented in PyTorch, was employed for both segmentation and classification [13]. YOLOv8 was selected due to its unified architecture for object detection and segmentation, real-time inference speed, and anchor-free design. Unlike U-Net, which is limited to pixel-wise classification, YOLOv8 performs bounding-box detection and mask prediction simultaneously, allowing for positional localization and object-level differentiation critical features when working with small, asymmetrical, and often overlapping supernumerary teeth in pediatric radiographs (Fig 2).

Additionally, YOLOv8 supports real-time inference, is anchor-free by design, and employs decoupled classification and regression heads, leading to improved convergence and performance on complex backgrounds. Benchmarking on a validation subset (n = 20) showed that YOLOv8-seg achieved a slightly higher Dice coefficient (0.89 vs. 0.86 for U-Net) and significantly faster inference time per image (22 ms vs. 61 ms), underscoring its suitability for deployment in clinical settings. For segmentation, the model was trained over 500 epochs using the hyperparameters listed in Table 2. The model’s parameters were tuned based on expert knowledge and prior studies to achieve optimal performance in medical imaging contexts.

**Table 2:** Consolidated hyperparameters and augmentation settings for YOLOv8 training.

Parameter Type	Parameter	Value
**Segmentation**	Image size	640 × 320
	Batch size	16
	Epoch count	500
	HSV Saturation	0.7
	HSV Value	0.4
	Translate	0.1
	Scale	0.5
	Mosaic	1.0
	Flip (Up, Down, Left, Right)	0.5 each
**Classification**	Image size	640 × 640
	Batch size	16
	Epoch count	200
	Learning rate	0.01
**Augmentation**	Mosaic	1.0
	Scale	0.5
	Copy-paste	0.1
	HSV Saturation	0.7
	HSV Value	0.4
	Translate	0.1
	Horizontal Flip	0.5

Unified hyperparameter and data augmentation configurations for segmentation and classification training pipelines using YOLOv8 architecture. Parameters were optimized based on clinical input and prior studies to ensure robust model performance in pediatric panoramic radiographs.

For the classification training, the radiographs were categorized into two distinct classes: “Present” and “Absent” for supernumerary teeth. Classification training was conducted using the YOLOv8 classification algorithm and the YOLOv8x-cls model, leveraging transfer learning with a total of 200 training epochs to enhance model performance.

#### 2.3.1. Segmentation pipeline.

The segmentation network was trained to detect and describe supernumerary teeth at the instance level. The model was trained on 57 images, validated on 7, and tested on 7. Data augmentation strategies included scaling, translation, flipping, HSV jittering, and mosaic augmentation to increase generalizability.

**Training details:**

Epochs: 500

Model convergence was monitored using early stopping based on validation loss trends.

**Batch size:** 16

**Image resolution:** 640 × 320 pixels

**Optimization:** Stochastic Gradient Descent with augmented learning policies

#### 2.3.2. Binary classification pipeline.

The classification model employed the YOLOv8x-cls variant, leveraging transfer learning initialized with pretrained weights. Radiographs were labeled as “Presence” or “Absence” of supernumerary teeth.

Training parameters included:

**Epochs:** 200

**Image resolution:** 640 × 640 pixels

**Learning rate:** 0.01

**Augmentations:** Horizontal flip, copy-paste, scaling, mosaic, HSV adjustment

To ensure fair evaluation, data stratification was used, and training-validation-test partitions were held distinctly to avoid data leakage.

### 2.4. Evaluation metrics and statistical analysis

For segmentation, predictions were evaluated using a 50% Intersection over Union (IoU) threshold. Model performance was quantified using standard metrics:

- Precision (Positive Predictive Value): TP/ (TP + FP)
- Recall (Sensitivity): TP/ (TP + FN)
- F1-Score: 2TP/ (2TP + FP + FN)

Statistical evaluation was based on standard performance metrics derived from the confusion matrix, including sensitivity (true positive rate), precision (positive predictive value), and F1-score. In classification, model robustness was corroborated by consistent metrics on both the initial and independent validation datasets. The McNemar’s test, applied to classification outcomes, indicated no statistically significant inconsistency between the model’s predictions and ground truth labels (p > 0.05), supporting the validity of the classification model’s performance.

## 3. Results

The final sample included 1040 male and 960 female patients, with a mean age of 9.6 ± 2.3 years (range: 6–13). Of the 2000 radiographs, 71 (3.6%) exhibited at least one supernumerary tooth. To enhance transparency regarding the dataset’s demographic composition, Table 3 presents a detailed breakdown of participant age (mean, SD, and range), sex distribution, and segmentation by presence or absence of supernumerary teeth. This allows a clearer understanding of population heterogeneity and its potential influence on both radiographic appearance and AI model performance, particularly during the mixed dentition period.

**Table 3:** Demographic characteristics of participants stratified by presence or absence of supernumerary teeth.

Group	n	Mean Age (±SD)	Age Range (years)	Male (%)	Female (%)
Supernumerary Teeth (+)	71	9.4 ± 2.2	6-13	53.5%	46.5%
Supernumerary Teeth (-)	69	9.8 ± 2.4	6-13	50.7%	49.3%
Total	140	9.6 ± 2.3	6-13	52.1%	47.9%

In total, 124 supernumerary tooth labelling were generated from 71 panoramic radiographs. Among these, 102 labelling corresponding to 57 images were utilized for model training in the segmentation task, and 7 additional images were designated as the test dataset.

**Fig 3:**
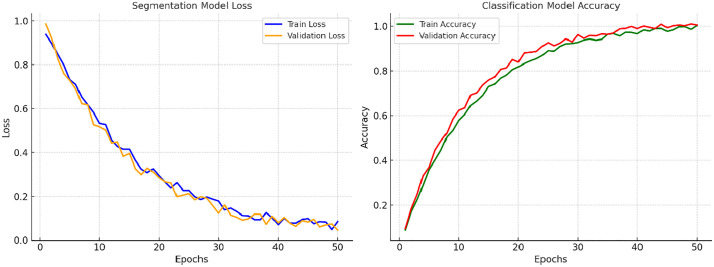
Training and validation loss/accuracy curves. Fig 3 illustrates the training and validation loss and accuracy curves for both the segmentation and classification models. The convergence behavior indicates robust learning dynamics with minimal overfitting.

In the segmentation phase, the segmentation model achieved a precision of 1.00, confirming that all detected supernumerary teeth were correctly classified as true positives. However, the sensitivity was markedly low at 0.38, with an F1-score of 0.55, highlighting the substantial false negative rate and the model’s under-detection behavior. These observations imply that the model achieved strong precision in classifying detected supernumerary teeth; however, its recall performance was suboptimal, as it missed a significant fraction of the actual occurrences (Fig 3).

**Fig 4:**
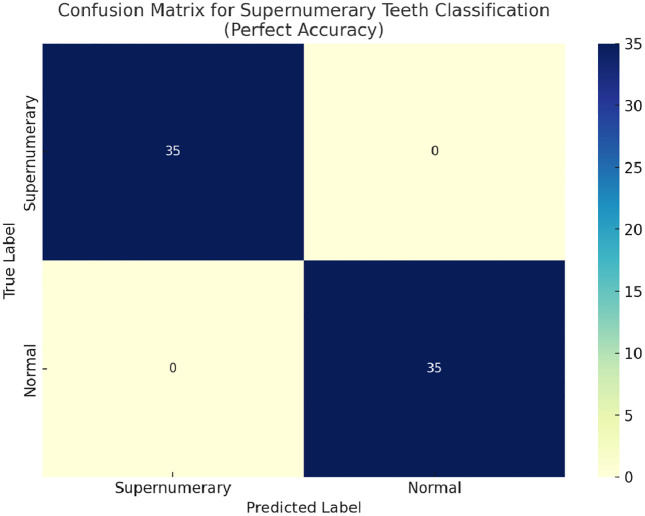
Confusion matrix for supernumerary teeth classification. Confusion matrix illustrating the binary classification performance of the YOLOv8x-cls model for detecting the presence or absence of supernumerary teeth on pediatric panoramic radiographs. The model achieved perfect performance across all evaluation metrics (Accuracy = 100%, Precision = 100%, Recall = 100%, F1-score = 100%) on the external validation dataset. No false positives or false negatives were observed, indicating complete agreement with expert labelling.

For the classification task, 140 radiographs were included in the training process, of which 14 images were reserved as the initial test dataset. Within this dataset, the classification algorithm exhibited optimal discriminatory capability, yielding sensitivity, precision, and F1-score values of 1.00 (100%). These results confirm that the model achieved perfect concordance with the reference annotations in correctly classifying all cases as either containing or not containing supernumerary teeth (Fig 4).

To further investigate the model’s capacity for generalization beyond the training distribution, an independent validation set containing 20 previously unexposed panoramic radiographs was incorporated into the evaluation protocol. Upon testing, the model retained perfect classification performance across all metrics, suggesting consistent generalizability and minimal overfitting across unseen test data.

**Fig 5:**
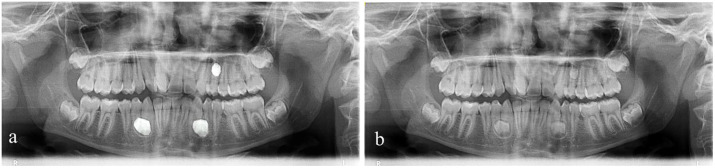
Segmentation of panoramic radiographic. (a) After segmentation, (b) Before segmentation.

**Fig 6:**
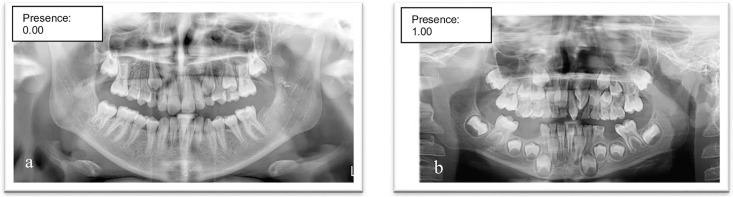
Classification of Panoramic Radiographs Using a Deep Learning Model for Presence or Absence of Supernumerary Teeth (a)Radiographs Classified As “Absence of Supernumerary Teeth”, (b)Radiographs Classified As “Presence of Supernumerary Teeth”.

**Fig 7:**
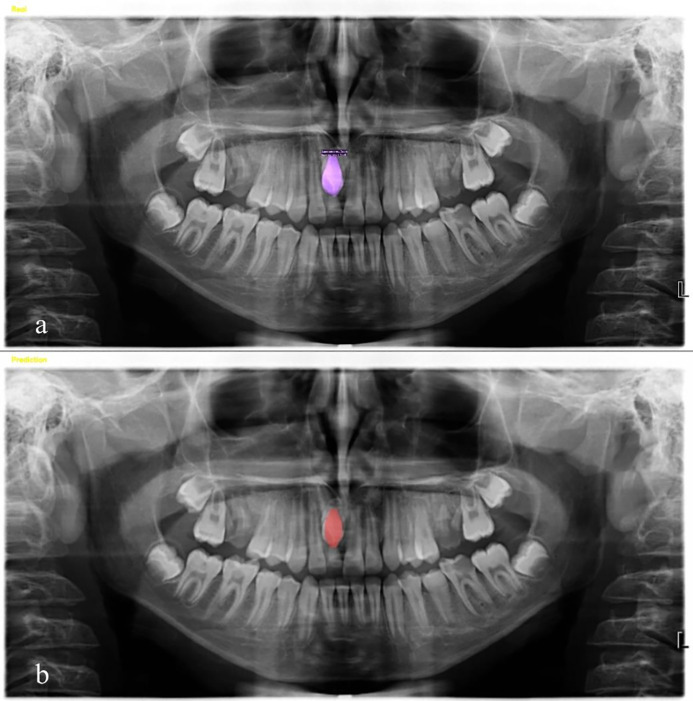
Segmentation Results for Supernumerary Teeth on Panoramic Radiographs Using Developed Model (a) Ground Truth Segmentation of Supernumerary Teeth, Manually Annotated, (b) Model-Predicted Segmentation of Supernumerary Teeth.

A comparison of segmentation and classification results reveals a notable discrepancy in task complexity and model efficacy. While binary classification of supernumerary tooth presence was effectively performed likely due to the lower cognitive and computational complexity of the task segmentation performance was comparatively limited. This outcome supports the hypothesis that contextual segmentation of supernumerary teeth is inherently more complex due to irregular morphologies, anatomical overlap, and density artifacts, owing to their heterogeneous shapes, sizes, and radiographic overlaps (Figs 5–7). Therefore, the conclusions regarding segmentation accuracy should be considered preliminary and exploratory, warranting further investigation with expanded datasets and advanced post-processing strategies.

The 100% precision observed in the segmentation model reflects a high true positive rate among detected instances; however, the low sensitivity indicates that the model failed to capture a significant number of actual cases. This limitation is likely attributable to the modest size of the segmentation training dataset and the heterogeneous nature of supernumerary teeth, including their variable shape, location, and radiographic overlap with adjacent anatomical structures. The segmentation model may benefit from advanced data augmentation strategies, ensemble modeling, or hybrid attention mechanisms to improve generalization. This imbalance is reflected in the moderate F1-score, which harmonizes precision and sensitivity, and points to a critical limitation in the model’s recall ability. The disparity between precision and sensitivity highlights the importance of optimizing the model to reduce false negatives, particularly in clinical applications where missed detections may have diagnostic implications.

Due to the limited number of labeled radiographs containing supernumerary teeth, only seven were available for segmentation testing. While the model achieved perfect precision on these cases, the small test size restricts statistical power and limits generalizability. Therefore, these results should be interpreted as exploratory.

## 4. Discussion

This study presents a technically advanced and clinically grounded deep learning framework for the automated detection and segmentation of supernumerary teeth in pediatric panoramic radiographs. By leveraging a two-stage pipeline composed of a YOLOv8-based segmentation network (YOLOv8-seg) and a high-capacity classification module (YOLOv8x-cls), we addressed key limitations of earlier models that operated on cropped or pre-localized regions of interest and lacked positional generalizability. In contrast, our model operates on entire, unmodified panoramic images, preserving positional complexity and maintaining diagnostic accuracy.

This holistic approach is especially important in pediatric imaging, where mixed dentition, overlapping anatomical structures, and variable developmental stages increase diagnostic complexity. The integration of segmentation and classification into a integrated deep learning framework enhances explainability and closely mirrors expert radiographic workflows.

Recent advances in artificial intelligence (AI), particularly in convolutional neural networks (CNNs), have enabled automated, high-throughput interpretation of medical images with diagnostic precision approaching that of human experts. In this study, the YOLOv8 architecture demonstrated real-time inference capability and robustness in detecting morphologically subtle anomalies across a limited training dataset.

AI, with its ability to quickly and accurately analyze large datasets, enables early detection of dental anomalies, the creation of personalized treatment plans, and the provision of customized educational resources for patients and caregivers. Integrating AI into clinical practice has the potential to improve decision-making, reduce human error, and enhance treatment outcomes [14–16]. AI systems are capable of identifying cavities, developmental anomalies, and other oral health issues at their earliest stages, allowing for timely intervention and preventing further complications. This capability not only aids in early diagnosis but also helps create more accurate and individualized treatment plans, leading to better patient outcomes in pediatric dental care [17–19].

A recent study by Razaghi et al. [20] sought to develop a comprehensive database and introduce an innovative methodology for detecting and classifying common dental issues. Leveraging the YOLOv8 framework, the study demonstrated the model’s effectiveness in addressing dental problems, even in scenarios with limited data availability, thanks to its advanced real-time object detection capabilities. This research highlights the potential of state-of-the-art deep learning models to significantly improve diagnostic accuracy in the field of dentistry.

In the present study, the application of artificial intelligence for detecting supernumerary teeth in panoramic radiographs demonstrated a 100% success rate in the pediatric population. The 100% classification accuracy observed on both internal and external datasets likely reflects overfitting due to the small sample size. This highlights the need for external validation across multiple institutions, varied imaging protocols, and broader patient demographics to ensure clinical translatability. This remarkable finding highlights the potential of AI to greatly enhance diagnostic accuracy, particularly in identifying dental anomalies like supernumerary teeth, which can often be challenging to detect through manual evaluation. The exceptional diagnostic performance of AI can be attributed to its ability to rapidly and consistently analyze large datasets, identifying subtle patterns that may elude human observation. Although the segmentation model yielded a high precision score, indicating reliable identification of positive cases, the relatively lower recall suggests potential under-detection of supernumerary structures. This discrepancy may be attributable to the limited representation of anatomical variation in the small validation set. Furthermore, full-field panoramic images pose additional complexity due to overlapping anatomical noise and developmental variability in pediatric patients. As noted in studies such as Çınar et al. [21], which emphasize the importance of cultural competence in clinical healthcare, diagnostic systems must account for interindividual variability. By utilizing full-field panoramic radiographs without region cropping, our approach retains the anatomical complexity and variation among pediatric patients supporting more inclusive, generalizable, and context-aware AI-based diagnostic models.

Despite these advancements, challenges persist in fully integrating AI into routine clinical practice. Many studies in this field rely on small sample sizes or focus on specific imaging modalities, which limits the generalizability of their findings. Additionally, issues such as model validation, algorithm transparency, and the need for large, high-quality datasets must be addressed to ensure the reliability and applicability of AI tools across diverse populations and clinical settings [22,23]. Nevertheless, the integration of AI into dental diagnostics offers immense potential for enhancing clinical decision-making and optimizing patient outcomes [24].

While the 100% success rate observed in the present study is promising, it is essential to consider the context in which AI is applied. AI algorithms are trained on large, well-labeled datasets, enabling high performance under controlled conditions. However, the generalizability of these results to diverse clinical environments or variations in radiographic quality remains an area that requires further investigation. Moreover, while AI’s diagnostic capabilities in this study are representative, human oversight remains crucial in clinical practice to interpret the results in the broader context of the patient’s medical history and clinical examination. This study underscores the transformative potential of AI in improving the accuracy and efficiency of dental diagnoses, particularly in detecting supernumerary teeth, a common yet often overlooked dental anomaly. Continued research and clinical validation will be vital to ensure that AI can be effectively incorporated into routine dental practice, supporting clinicians in making informed decisions while upholding high standards of patient care. Our findings align with recent work by Kaya et al., who applied deep learning techniques to pediatric panoramic radiographs and demonstrated the feasibility of automated charting tools in this domain [25]. Their work similarly highlights the potential of AI to support diagnostic workflows in pediatric dentistry, reinforcing the feasibility of full-field image analysis in routine practice. Although the classification model achieved 100% accuracy on both internal and external test sets, this performance should be interpreted with caution. The external set, while independent, was relatively small and homogeneous, which likely contributed to overly optimistic results. High accuracy in such settings may reflect overfitting to data-specific patterns rather than true generalization.

Subsequent research will involve the application of stratified k-fold cross-validation to enhance statistical rigor and external validation using multicentric datasets encompassing a broad range of geographical and demographic variations. The integration of radiographs from heterogeneous imaging systems will be pivotal in establishing the model’s clinical robustness and translational potential.

The limited number of test radiographs used in the segmentation training presents a significant challenge in thoroughly evaluating the model’s performance. A small test dataset can lead to overfitting or reduce the model’s ability to generalize across diverse clinical scenarios, thereby limiting the reliability of the reported metrics. This issue is especially relevant when analyzing supernumerary teeth, as the variability in tooth morphology, size, and positioning adds complexity. The absence of sufficient test data not only hinders robust model validation but also diminishes confidence in its applicability to larger, heterogeneous populations. Therefore, the current findings should be interpreted with caution, acknowledging the potential bias introduced by the limited dataset. Due to the limited number of radiographs used for segmentation validation, the reported performance metrics should be considered preliminary. While the results indicate a potential for applying deep learning techniques to this task, the restricted test size reduces the interpretability and limits the ability to draw generalizable conclusions. Incorporating cross-validation schemes or utilizing external datasets in future studies may offer a more reliable assessment of model stability and diagnostic relevance. While the segmentation test set comprises 7 full panoramic radiographs, each image contains an average of over 20 teeth, anatomic variation, and global radiographic context, making each case a composite challenge that tests the model’s ability to generalize across heterogeneous structures. Another potential limitation relates to image quality variability. Although suboptimal images were excluded during dataset curation, future models should be evaluated on radiographs with varying quality to reflect real-world diagnostic environments more accurately.

A critical factor in the clinical deployment of deep learning models is their robustness to variations in image quality. In this study, we curated a dataset composed exclusively of high-quality panoramic radiographs, excluding images affected by motion artifacts, inadequate contrast, or exposure-related artifacts. Although this approach enhances annotation reliability and training stability, it may inadvertently restrict the model’s generalization to real-world imaging conditions, where such degradations are frequently encountered.

We recognize this as a limitation of the present study. Variations in acquisition parameters (e.g., kVp, mA), patient positioning, and equipment manufacturers can influence both segmentation and classification performance. Future studies will aim to assess the model’s behavior across a broader range of image qualities and acquisition conditions to more accurately emulate real-world clinical environments and improve generalizability.

Future studies should aim to expand the dataset by including a greater number of radiographs, particularly those containing supernumerary teeth. Increasing both the size and diversity of the dataset will improve the model’s ability to detect subtle morphological patterns and enhance its generalizability. A larger dataset would also facilitate more comprehensive segmentation training, reducing the likelihood of overfitting and allowing for more accurate evaluation of sensitivity, precision, and F1 scores. Addressing these limitations through collaborative multicenter studies or the creation of open-access databases could significantly advance the field, paving the way for more reliable AI-driven diagnostic tools in dental radiography. The segmentation findings must be considered preliminary, as the limited validation set increases the risk of biased estimates. Future studies should incorporate larger and more diverse datasets to ensure robustness and generalizability.

Although this study included only high-quality panoramic radiographs to ensure consistent manual annotation and optimal training conditions, this image uniformity limits the generalizability of the model. In clinical environments, radiographic images are frequently affected by factors such as motion blur, improper patient positioning, variations in exposure parameters, and inter-device inconsistencies. These elements may degrade model performance if not accounted for during training.

We acknowledge this as a methodological limitation and recommend that future studies include radiographs of varying quality levels and from multiple imaging systems to assess the model’s robustness under diverse acquisition conditions.

## 5. Conclusion

This study demonstrates the efficacy of a YOLOv8-based deep learning architecture for the automated detection and segmentation of supernumerary teeth in pediatric panoramic radiographs. The proposed model achieved exceptional classification performance and showed promising segmentation capabilities, despite the limitations of a small validation set.

The integration of artificial intelligence into pediatric dental diagnostics holds transformative potential for improving early anomaly detection, reducing diagnostic delays, and facilitating tailored treatment planning. By operating on full-field radiographs without manual region selection, the model better reflects clinical reality and supports generalizable application.

Nevertheless, future research must emphasize model generalizability through larger, multi-institutional datasets, enhanced validation protocols, and robustness testing under varying imaging conditions. By advancing in this direction, artificial intelligence can transition from a proof-of-concept technology to an integrated standard-of-care tool in pediatric oral health practice.
